# Multicentre evaluation of a selective isolation protocol for detection of *mcr*‐positive *E. coli* and *Salmonella* spp. in food‐producing animals and meat

**DOI:** 10.1111/lam.13717

**Published:** 2022-04-21

**Authors:** Agnès Perrin‐Guyomard, Sophie A. Granier, Jannice Schau Slettemeås, Muna Anjum, Luke Randall, Manal AbuOun, Natalie Pauly, Alexandra Irrgang, Jens Andre Hammerl, Jette Sejer Kjeldgaard, Anette Hammerum, Alessia Franco, Magdalena Skarżyńska, Ewelina Kamińska, Dariusz Wasyl, Cindy Dierikx, Stefan Börjesson, Yvon Geurts, Marisa Haenni, Kees Veldman

**Affiliations:** ^1^ French Agency for Food, Environmental and Occupational Health & Safety (ANSES) Fougères Laboratory Fougères France; ^2^ Norwegian Veterinary Institute Oslo Norway; ^3^ 16232 Animal and Plant Health Agency Addlestone UK; ^4^ German Federal Institute for Risk Assessment Berlin Germany; ^5^ 5205 Technical University of Denmark Lyngby Denmark; ^6^ 4326 Statens Serum Institut Kobenhavn Denmark; ^7^ 18353 Istituto Zooprofilattico Sperimentale del Lazio e della Toscana Lazio Italy; ^8^ National Veterinary Research Institute Pulawy Poland; ^9^ 10206 National Institute for Public Health and the Environment Bilthoven the Netherlands; ^10^ National Veterinary Institute (SVA) Sweden and Public Health Agency of Sweden Uppsala Sweden; ^11^ 4507 Wageningen Bioveterinary Research Lelystad the Netherlands; ^12^ Lyon University – French Agency for Food, Environmental and Occupational Health & Safety (ANSES) Lyon Laboratory Lyon France

**Keywords:** animals, Colistin resistance, *E. coli*, *mcr*, *Salmonella*, screening

## Abstract

This study was conducted to evaluate the performance of a screening protocol to detect and isolate *mcr*‐positive *Escherichia coli* and *Salmonella* spp. from animal caecal content and meat samples. We used a multicentre approach involving 12 laboratories from nine European countries. All participants applied the same methodology combining a multiplex PCR performed on DNA extracted from a pre‐enrichment step, followed by a selective culture step on three commercially available chromogenic agar plates. The test panel was composed of two negative samples and four samples artificially contaminated with *E. coli* and *Salmonella* spp. respectively harbouring *mcr*‐*1* or *mcr*‐*3* and *mcr‐4* or *mcr*‐*5* genes. PCR screening resulted in a specificity of 100% and a sensitivity of 83%. Sensitivity of each agar medium to detect *mcr*‐positive colistin‐resistant *E. coli* or *Salmonella* spp. strains was 86% for CHROMID^®^ Colistin R, 75% for CHROMagar^TM^ COL‐*APSE* and 70% for COLISTIGRAM. This combined method was effective to detect and isolate most of the *E. coli* or *Salmonella* spp. strains harbouring different *mcr* genes from food‐producing animals and food products and might thus be used as a harmonized protocol for the screening of *mcr* genes in food‐producing animals and food products in Europe.

## Graphical abstract

**Significance and impact of the study:** We performed a multicentre study to evaluate a protocol for the detection of *mcr*‐carrying isolates combining selective pre‐enrichment, PCR and cultivation on commercial chromogenic selective agars. Adding a multiplex PCR step to detect *mcr* genes after the pre‐enrichment step can both save time and reduce costs by discarding negative samples. The performance of the method was influenced by the selective agar used and by the *mcr* variants to be detected. We propose a robust protocol that could be used in the frame of a harmonized European screening of *mcr*‐positive *E. coli* and *Salmonella* spp. in food‐producing animals and food products.

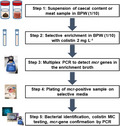

## Introduction

Colistin has been extensively used in veterinary medicine for decades, mostly to prevent gastrointestinal diseases in piglets, calves and poultry (Catry *et al*. 2015). In human medicine, the use of colistin has long been very limited related to its potential toxicity (Koch‐Weser *et al*. 1970), but colistin has regained interest as a treatment option for life‐threatening infections caused by multidrug resistant (MDR) Gram‐negative bacteria (Nørgaard *et al*. 2019). Before 2015, the only described mechanisms for colistin resistance were chromosomal mutations in genes involved in the modification of the lipopolysaccharide charge of the outer cell membrane (Olaitan *et al*. 2014). However, in 2015, Liu *et al*. (2016) discovered the first transferable colistin‐resistance gene, called *mcr‐1*. Numerous publications showed the worldwide dissemination of *mcr‐1* among *Enterobacterales* (Xavier *et al*. 2016), with most of the described *mcr‐1*‐positive *Enterobacterales* being of animal origin. Since this first description, nine new *mcr* genes (*mcr‐2* to *mcr‐10*) and their diverse variants have been identified (Wang *et al*. 2020). The identification of the transferable colistin‐resistance genes and the increasing use of colistin prompted the WHO to include this antibiotic as a highest priority critically important antimicrobial in humans (WHO Advisory Group on Integrated Surveillance of Antimicrobial Resistance (AGISAR) 2019). The prevalence of *mcr*‐*1* carriers isolates was much higher in food‐producing animals and food sources than in humans (Carnevali *et al*. 2016; Elbediwi *et al*. 2019). This observation raised questions on the veterinary use of colistin and the potential contribution of food‐producing animals to colistin‐resistance in humans (Skov and Monnet 2016).

Based on phenotypic data, the European monitoring of antimicrobial resistance in zoonotic and commensal bacteria indicated that colistin resistance is still uncommon in *Salmonella* spp. and *E. coli* isolated from food‐producing animals (EFSA and ECDC 2021). However, the major limitation of these results is the absence of detection of *mcr*‐positive *Enterobacterales* on selective media. In the meantime, an increase in *mcr*‐positive isolates from healthy livestock has been observed in some countries (Zając *et al*. 2019; Oh *et al*. 2020), while metagenomic analysis revealed that *mcr* gene variants can be detected in high relative abundances from raw municipal wastewater in Germany (Kneis *et al*. 2019).

To date, several different screening protocols have been developed to detect the presence of *mcr* genes from both clinical samples and isolated strains (Bontron *et al*. 2016; Nordmann *et al*. 2016; Abdul Momin *et al*. 2017; Bardet *et al*. 2017; Li *et al*. 2017; Rebelo *et al*. 2018; García‐Fernández *et al*. 2019). However, the lack of reliable and comparable consensus methodology prompted us to evaluate, harmonize and implement a selective method for the detection of *mcr*‐positive *Salmonella* spp. and *E. coli* from the food chain. To this end, the One Health European Joint Programme (https://onehealthejp.eu/) IMPART consortium (https://onehealthejp.eu/jrp‐impart/) gathering both European veterinary and public health laboratories working on antimicrobial resistance was set up to develop *inter alia* a sensitive screening assay for the detection of *mcr*‐positive colistin‐resistant *Enterobacterales*. This study thus describes the multicentre evaluation of a harmonized screening method for the selective detection of *mcr‐1* to *mcr‐5*‐positive *E. coli* and *Salmonella* spp. in animal caecal content and meat samples.

## Results and Discussion

### Validation of the selective media

The colistin‐susceptible reference strain *E. coli* ATCC 25922 did not grow on any of the selective agar plates tested, while all laboratories reported growth of their respective positive control strain on the CHROMID^®^ Colistin R agar and the CHROMagar^TM^ COL‐*APSE* (Table 1), leading to a 100% sensitivity. Contrarily, only seven out of eleven laboratories reported growth of their positive control strains on the COLISTIGRAM agar (Table 1), giving a 63·6% sensitivity of the medium. The four laboratories that reported no growth on COLISTIGRAM used *E. coli* or *Salmonella* spp. strains presenting colistin minimum inhibitory concentration (MIC) values between 4 and 8 mg L^−1^ (Table 1). These strains should have grown on the agar as this medium contains colistin concentration equivalent to 2 mg L^−1^ according to the manufacturer instructions. The reason for this lack of growth could not be explained, so this medium seemed to be less suitable for the detection of colistin‐resistant *Salmonella* spp. and *E. coli*.

**Table 1:** Quality control results of each selective medium

Positive control used (COL MIC mg L^−1^)	Laboratory code										
	A	B	C	D	E	F	G	H	I	J	K
	*Escherichia coli mcr‐4* (2)	*E. coli mcr‐1* (2)	*E. coli mcr‐1* (4)	*E. coli mcr‐2* (4)	*E. coli mcr‐1* (4)	*E. coli mcr‐2* and *S*. Paratyphi *mcr‐5* (4)	*E. coli mcr‐1* (8)	*E. coli mcr‐1* (4)	*S*. Typhimurium *mcr‐5* (4)	*E. coli mcr‐3* (4)	*E. coli mcr‐3* (4‐8)
CHROMID^®^ Colistin R	+	+	+	+	+	+	+	+	+	+	+
CHROMagar^TM^ COL‐*APSE*	+	+	+	+	+	+	+	+	+	+	+
COLISTIGRAM	+	+	+	−	−	−	+	+	−	+	+

(+) indicates that strains grew on the selective medium (−) indicates that strains did not grow on the selective medium. COL MIC: colistin MIC.

### Performance of the PCR to detect *mcr*‐positive samples

All participants received the same panel of samples under cold conditions and within a maximum of 24 h after shipping. With the exception of one lab (Lab F) which kept the samples frozen before processing, all laboratories started the analysis soon after arrival. Since a freezing step might alter the load of resistant bacteria that were inoculated, results of laboratory F were excluded from further analysis.

All negative samples in the panel were correctly identified, resulting in a 100% specificity (Table 2). Among positive samples, none of the participants was able to detect the *mcr‐5* gene by PCR from the meat sample spiked with the *mcr‐5 Salmonella* Schwarzengrund. Homogeneity and stability tests performed by the organizing laboratory confirmed that neither the *mcr*‐*5* gene nor the *mcr*‐*5 Salmonella* Schwarzengrund strain could be detected by PCR or plating. This sample was thus excluded from the performance analysis. In the three remaining *mcr*‐positive samples, (i) the *mcr‐1* strain spiked in pig caecal sample was detected by all participants, (ii) the *mcr‐3* strain spiked in pig caecal sample was correctly detected by all but one participant, and (iii) the *mcr‐4* strain spiked in turkey meat was correctly identified by only six out of ten participants. This resulted in an overall PCR sensitivity of 83%. The PCR sensitivity was however higher for pig caecal samples (95%) than for turkey meat samples (80%). More samples would be needed to consolidate this observation, but it is possible that the initial inoculum of 10^2^ CFU per g of *Salmonella* in meat samples could have been decreased or inhibited during the enrichment step by a growth competition of the endogenous flora of the meat, leading to false negative results in the PCR step. Indeed, previous studies have shown that at least 10^3^ *Salmonella* per mL must be present in broth‐enriched chicken meats to yield positive PCR results and that natural background microflora in poultry meat could limit the growth of foodborne pathogens (Soumet *et al*. 1994; Li and Mustapha 2002; Croci *et al*. 2004; Kanki *et al*. 2009; Lardeux *et al*. 2015). A limitation of the study is also that the PCR protocol used was initially validated on DNA extracted from bacterial pure cultures, while DNA used here was extracted from a complex matrix that may interfere with the effectiveness of the PCR reaction. Several publications have also reported that the presence of food constituents, such as organic and phenolic compounds, glycogen, fats and Ca^2+^, can inhibit DNA polymerase activity during PCR amplification (Rossen *et al*. 1992; Schrader *et al*. 2012; Rouger *et al*. 2017).

**Table 2:** PCR screening results according to the participant and the samples

Sample	*mcr*status	Laboratory code											Score	Performance	
		A	B	C	D	E	*F*	G	H	I	J	K		Specificity	Sensitivity
Pig caecal content	*mcr* negative (sample 1)	−	−	−	−	−	−	−	−	−	−	−	10/10	100%	83%
	*mcr‐1* positive (sample 2)	+	+	+	+	+	−	+	+	+	+	+	10/10		
	*mcr‐3* positive (sample 3)	+	+	+	+	+	−	+	+	+	+	−	9/10		
Turkey meat	*mcr* negative (sample 4)	−	−	−	−	−	−	−	−	−	−	−	10/10		
	*mcr‐4* positive (sample 5)	−	+	+	+	−	+	+	+	+	−	−	6/10		
	*mcr‐5* positive (sample 6)	−	−	−	−	−	−	−	−	−	ND	−	NA		

(−) indicates a negative result with no mcr amplification; (+) indicates a positive result with the amplification of the target mcr gene.

Results of participant F are not included in the performance analysis.

ND, not determined; NA, data not available.

### Performance of the selective media to isolate *mcr*‐positive bacteria

The performance of each selective agar medium for the detection of *mcr*‐positive isolates from pre‐enriched meat and caecal samples is presented in Table 3. The *mcr‐1*, *mcr‐3* and *mcr‐4* positive colistin‐resistant *E. coli* or *Salmonella* spp. strains were isolated 25 times out of 29 (86%) on CHROMID^®^ Colistin R, 18 times out of 24 (75%) on CHROMagar^TM^ COL‐*APSE* and 16 times out of 23 (70%) on COLISTIGRAM, respectively. No statistically significant differences in growth performance was observed between the media (*P* = 0·03). Our results on CHROMID^®^ Colistin R were consistent with the results of García‐Fernández *et al*. (2019), where sensitivity of this medium to isolate colistin‐resistant *Enterobacterales* varied from 87·1 to 89·3% when streaking rectal swabs or stool samples respectively.

**Table 3:** Plating results on each selective medium according to the participant and the samples

													Selective medium																							
	CHROMID^®^ Colistin R												CHROMagar^TM^COL−*APSE*												COLISTIGRAM											
Lab code	A		B	C	D	E	*F*	G	H	I	J	K	A		B	C	D	E	*F*	G	H	I	J	K	A	B	C	D	E	*F*	G	H	I	J	K
Sample 2 (*mcr‐1* +)	+		+	+	+	+	−	+	+	+	+	+	+		+	ND	+	+	−	+	+	ND	+	+	+	+	ND	+	+	−	+	+	ND	+	+
Sensitivity	100%												100%												100%											
Sample 3 (*mcr‐3* +)	+	+		+	+	+	−	+	+	+	+	−	+		+	ND	+	+	−	+	+	ND	+	−	+	+	ND	+	+	−	+	+	ND	+	−	
Sensitivity	90%												88%												88%											
Sample 5 (*mcr‐4* +)	−	+		+	−	ND	+	+	+	+	−	+	–		+	−	−	ND	+	+	−	ND	−	+	−	−	−	−	ND	+	+	−	ND	ND	−	
Sensitivity (%)	67%													38%												14%										
Global sensitivity	86%													75%												70%										

(+) indicates that the expected strain was isolated and confirmed phenotypically and genotypically, (−) indicates that the expected strain could not be isolated on the selective medium or could not be confirmed as *mcr*‐positive *Escherichia coli* or *Salmonella* spp., ND: not determined (Participant C: pure culture of typical colonies, *E. coli* for bacterial identification but neither phenotypic nor genotypic confirmation; Participant E: no plating because of PCR negative results; Participant I: pure culture of typical colonies from samples 2 and 3, mixed culture of typical colonies from sample 5 but neither bacterial identification, phenotypic nor genotypic confirmation; Participant J : No results). Results of participant F are not included in the performance analysis.

All but one participant (Lab G) isolated and identified the expected *mcr*‐positive bacteria according to their in‐house protocol (Table S1) and reported the expected MIC value plus or minus one dilution step, which is within the expected variation of the method. The *mcr* genes detected in the isolated strains confirmed the results obtained in the PCR after the enrichment step.

The sensitivity of the selective agar media for the detection of colistin‐resistant *Enterobacterales* varied depending on the bacteria‐gene combinations and matrices. When considering caecal sample results only (samples 2 and 3), the three selective media gave very similar results, and the slight difference in the overall performance (94%–95%) was exclusively due to the fact that two laboratories only reported complete results on CHROMID^®^ Colistin R. All participants were able to isolate the *mcr‐1‐*positive *E. coli* originating from caecal samples on COLISTIGRAM (also known as SuperPolymyxin agar), which is coherent with what Nordmann *et al*. observed with stool samples from humans volunteers (Nordmann *et al*. 2016). CHROMagar^TM^ COL‐*APSE* had only been validated using bacterial pure cultures where it reached similar performances as COLISTIGRAM (Abdul Momin *et al*. 2017); our results were coherent with this study and further indicated that CHROMagar^TM^ COL‐*APSE* is suitable to detect *mcr‐1‐* or *mcr*‐*3‐*positive *E. coli* originating from caecal samples.

For meat results, only sample 5 (artificially contaminated with *mcr‐4*) can be considered, since in coherence with results of the PCR step, none of the seven out of 10 participants who plated the enrichment broth from sample 6 detected the *mcr*‐*5 S*. Schwarzengrund strain on selective agars. Considering sample 5, CHROMID^®^ Colistin R presented a better sensitivity to detect colistin‐resistant strains harbouring *mcr‐4* compared with CHROMagar^TM^ COL‐*APSE* and COLISTIGRAM. To our knowledge, this is the first experiment using selective media to isolate colistin‐resistant strains from meat samples, so it is difficult to state whether this difference in performance is due to the medium, the gene (*mcr‐4*) or the bacteria (*Salmonella*). A possible explanation may reside in a recent study analysing the fitness cost of bacteria carrying *mcr‐1* to *mcr‐5*, where the growth of *mcr‐4* and *mcr‐5* strains in broth culture was significantly inhibited in the log phase when colistin was added in the medium (Li *et al*. 2021).

All participants reported growth of mixed flora from meat samples with typical and non‐typical colonies, which both increase the laboratory work and the difficulty to distinguish non‐chromogenic *Salmonella* spp. among background flora in meat samples. Isolated colonies were mostly identified as *Hafnia alvei* or *Serratia liquefaciens*, *Enterobacterales* known to be intrinsically resistant to colistin and present in meat samples (Odoi *et al*. 2021). These *Enterobacterales* could have masked the *mcr*‐carrying colistin‐resistant strains used for spiking of the meat samples, which is a second possible explanation for the reduced performance of the selective media to isolate *mcr*‐positive bacteria. Chromogenic agars were used in this protocol to facilitate the presumptive identification of colistin‐resistant *Enterobacterales,* but these agars have conversely some limitations that could have decreased the performance of the media. Indeed, several participants reported that reading the COLISTIGRAM was rather challenging. Colonies were usually small and the specific ones were hard to distinguish after 18–24 h incubation at 37°C, which could explain the lower performance of this medium. As in‐house preparation could add biases in growth results, preparation of CHROMagar^TM^ COL‐*APSE*, which is not commercially available as ready‐to‐use, was prepared centrally and sent to all participants. However, colistin added in the selective medium can be higher than the colistin MIC of some *mcr*‐harbouring strains, which results in missing target strains (Börjesson *et al*. 2020). In addition, species intrinsically resistant to colistin may grow on these selective media making reading difficult. Moreover, some bacterial strains may not produce the expected colour of the presumptive isolate as described in the manufacturer’s instructions. Indeed, in our study, the *mcr*‐*1*‐positive *E. coli* strain appeared colourless when streaked on CHROMID^®^ Colistin R, whereas pink colour was expected for this bacterial specie (Table S3). Conversely, the *mcr*‐*5*‐positive *S*. Schwarzengrund pure culture appeared pink on the same medium, whereas colourless colonies were expected. It is therefore important to emphasize that the identification of presumptive colistin‐resistant isolates must always be confirmed using phenotypic and/or genotypic methods.

In conclusion, we propose a practical, sensitive and specific harmonized protocol combining a PCR targeting *mcr* genes and a selective isolation step to monitor the prevalence of colistin‐resistant *E. coli* and *Salmonella* spp. from healthy animal caecal content and their derived meat. This combined methodology allows discarding PCR negative samples and focusing only on PCR positive ones. Given the number of samples to be analysed for active screening purposes, this pre‐screening PCR has an important added value to save time and consumables. One limitation of this trial is that only *mcr‐1* to *mcr‐5* genes were tested. Even though *mcr‐1* and *mcr‐3* are the most common colistin‐resistant genes detected in Europe, introducing the detection of other relevant *mcr* genes or variants might be of interest (Borowiak *et al*. 2020). The second step on selective chromogenic medium is a suitable screening tool to quickly isolate presumptive strains displaying acquired colistin‐resistance from animal samples. Generally, CHROMID^®^ Colistin R gave better results compared with CHROMagar^TM^ COL‐*APSE* and COLISTIGRAM. Even though additional trials are needed to extend the evaluation of the methodology to include more diverse *mcr*‐positive strains and matrices of different origins, our protocol could be advantageously used in the frame of a harmonized European screening of *mcr*‐positive *E. coli* and *Salmonella* spp. in food‐producing animals and food products.

## Materials and methods

### Participants

In total, 12 veterinary and/or public health laboratories (11 participants and one organizer) from nine countries volunteered in the multicentre evaluation study conducted in 2019 (Table 4). Participants filled their results obtained from quality controls, PCR and plating for each sample in an excel file sent by e‐mail to the organizing laboratory.

**Table 4:** Participating laboratories of the study

No.	Participating laboratory	Country
1	Anses, Fougères Laboratory	France (organizer)
2	Anses, Lyon Laboratory	France
3	Norwegian Veterinary Institute (NVI)	Norway
4	Animal and Plant Health Agency (APHA)	United Kingdom
5	German Federal Institute for Risk Assessment (BfR)	Germany
6	Technical University of Denmark (DTU)	Denmark
7	Statens Serums Institut (SSI)	Denmark
8	Istituto Zooprofilattico Sperimentale del Lazio e della Toscana “M. Aleandri” (IZSLT)	Italy
9	Państwowy Instytut Weterynaryjny (PIWET)	Poland
10	National Institute for Public Health and the Environment (RIVM)	The Netherlands
11	Veterinary Institute (SVA)	Sweden
12	Wageningen Bioveterinary Research (WBVR)	The Netherlands

### Bacterial isolates

Four *Enterobacterales* strains with acquired colistin resistance were used to spike samples from food‐producing animals or food products (Table 5). Two *E. coli* harbouring *mcr*‐*1* and *mcr*‐*3*, respectively, and two *Salmonella* spp. harbouring *mcr*‐*4* and *mcr*‐*5*, respectively, were included in the panel. The *mcr* genes were confirmed by PCR and whole‐genome sequencing (WGS) (Rebelo *et al*. 2018). Colistin‐susceptibility of the isolates was determined by broth microdilution (Thermo Scientific^TM^, Sensititre^TM^, Thermo Fisher, Dardilly, France). *Escherichia coli* ATCC 25922 was used as a negative control strain by all participants, while each participating laboratory used its own *mcr‐1* to *mcr‐5*‐producing *E. coli* or *Salmonella* spp. control strains to validate each selective agar (Table 1).

**Table 5:** Composition and characteristics of the panel of samples sent to participating laboratories

Sample		Spiked species			
		Name	Colistin MIC (mg L^−1^)	*mcr* gene	Reference
1	Pig caecal content	–	–	–	–
2	Pig caecal content	*E. coli* 15F001279	4	*mcr‐1*	Rebelo *et al*. (2018)
3	Pig caecal content	*E. coli* 15F001211	4	*mcr‐3*	Rebelo *et al*. (2018)
4	Turkey meat	–	–	–	–
5	Turkey meat	*Salmonella* 4,12:I 15Q004074	4	*mcr‐4*	Rebelo *et al*. (2018)
6	Turkey meat	*Salmonella* Schwarzengrund S12LNR3592	8	*mcr‐5*	Webb *et al*. (2016)

### Panel samples preparation

Previous experiments were performed to test the detection of *mcr*‐producing isolates from various combination of bacterial host and caecal content or meat samples from turkey, pig, calf or broiler on selective agar media containing colistin after enrichment with or without colistin. As no significant influence of bacteria–gene combinations and matrices was observed, meat from turkeys and caecal content from pigs were used for spiking the target strains in this ring trial (data not shown). In order not to overload the partner lab activities during the trial week, the IMPART consortium determined that six samples to be distributed to each lab was the reasonable goal to achieve. As *mcr‐1* and *mcr‐3* are frequently identified in *E. coli* originating from food‐producing animals (Perrin‐Guyomard *et al*. 2016; Hernández *et al*. 2017; Rebelo *et al*. 2018), and as these target bacteria–gene combination were easily available in our collections, *E.coli* harbouring *mcr‐1* or *mcr‐3* were spiked in pig caecal contents. As *mcr‐4* and *mcr‐5* were described and available to our IMPART consortium in *Salmonella* spp. isolated from animal origin including meat (Webb *et al*. 2016; Rebelo *et al*. 2018), these target bacteria–gene combinations were spiked in meat samples.

### Preparation of the matrices

Meat originated from specific pathogen free turkey raised in Anses farm and pig caecal content was collected at slaughterhouses in the context of the French antimicrobial surveillance programme. Each matrix batch was checked for the absence of acquired colistin resistance. A 1 : 10 dilution of caecal content and minced meat sample in buffered peptone water (BPW) supplemented with colistin (2 mg L^−1^) were incubated overnight (O/N) at 37°C. An *mcr*‐based multiplex PCR (Rebelo *et al*. 2018) was applied on the DNA of the overnight suspension (DNeasy blood and tissue kit, Qiagen, Courtaboeuf, France). A loop of 10 *µ*l of the overnight suspension was spread simultaneously on MacConkey agar (BD, Le Pont de Claix, France) containing colistin (2 mg L^−1^). Batches with negative PCR results and non‐typical acquired colistin‐resistant colonies were kept frozen (−20°C) until being artificially contaminated.

### Preparation of the inoculum

Strains were inoculated on blood agar and incubated O/N at 37°C. Prior to spiking, 0·5 McFarland (=approximately 10^8^ CFU per mL) bacterial suspensions were prepared in 0·9% NaCl solution using fresh colonies.

### Preparation of samples

The bacterial suspensions were diluted and added to the non‐contaminated matrices to obtain an arbitrary final concentration of 10^2^ CFU per g in the samples. Inoculated samples were homogenized and aliquoted 1 ± 0·1 g for caecal content and 10 ± 0·1 g for meat. One sample of each non‐contaminated matrix was used as a negative control. The aliquots were stored at 4°C before being shipped to all participants 72 h after preparation.

### Shipping of samples

Each panel of samples, as well as selective agar plates, was shipped to the participants in compliance with UN3373 regulations at 4°C. The participating laboratories received the samples with a unique code indicated on each sample. Analysis should be initiated immediately at arrival of the samples.

### Quality control checks

Homogeneity tests were performed for all samples with the methodology process of the study (Figs S1 and S2). For each sample, homogeneity was tested by PCR on three aliquots and by plating four aliquots in duplicate on CHROMID^®^ Colistin R (bioMérieux, Marcy‐L'Etoile, France) randomly selected from the positive test samples and by plating three aliquots in duplicate randomly selected from the negative test samples. The identity of sub‐cultured relevant isolates was confirmed by MALDI‐TOF (VITEK‐MS, bioMérieux, Marcy‐L'Etoile, France). The stability was tested using the same methodology on three aliquots randomly chosen among positive test samples. Plating was performed in duplicate on the three selective chromogenic media CHROMID^®^ Colistin R (bioMérieux,Marcy‐L'Etoile, France), CHROMagar^TM^ COL‐*APSE* (Mast Diagnostic, Amiens, France) and COLISTIGRAM (Kitvia, Labarthe Inard, France). The identity of sub‐cultured relevant isolates was confirmed by MALDI‐TOF (VITEK‐MS, bioMérieux, BrukerMarcy‐L'Etoile, France). Stability was assessed on the day of shipment (data obtained in the homogeneity study) and 1 day after reception and analysis of the samples by the participating laboratories. The results of stability testing were compared with those from the homogeneity tests (day 0). Results are presented in Table S2.

### Methodology process

The methodology process combined a PCR to readily exclude negative samples followed by a selective plating of positive ones as suggested previously (Osei Sekyere 2019). The selective agar plates were chosen if they were ready‐to‐use commercially available on European soil at the time of the trial. In 2019, we identified three products on the market: CHROMID^®^ Colistin R (bioMérieux, Marcy‐L'Etoile, France), CHROMagar^TM^ COL‐*APSE* (Mast Diagnostic, Amiens, France) and COLISTIGRAM (Kitvia, Labarthe Inard, France).

The workflow of the methodology is presented in Figs S1 and S2. A 1:10 dilution of caecal content and minced meat samples was processed in BPW. An initial culture of 3 h ± 1 h at 37°C was performed to allow bacterial flora moving from a dormant state in samples stored at 4°C to growth conditions. After mixing gently, 1 ml of pre‐enrichment culture was added to 9 ml of BPW supplemented with two discs of colistin 10 *µ*g in a polypropylene or glass tube. These enrichments were incubated at 37°C for 18–24 h. Each laboratory performed its own routine multiplex *mcr* PCR on DNA extracts from the overnight enrichment broth suspension (Table S1). Each enrichment broth from *mcr*‐positive PCR samples stored at 4°C was streaked on CHROMID^®^ Colistin R, CHROMagar^TM^ COL‐*APSE* and COLISTIGRAM. Inoculated media were incubated at 37°C for 18–24 h. Based on reading interpretation of each manufacturer (Table S3), a minimum of three relevant colonies were re‐isolated by sub‐culturing on a new selective agar plate and incubated at 37°C for 18–24 h. Species identification on sub‐cultured isolates was performed using the method preferred in each participating laboratory (Table S1). Colistin‐resistance was confirmed phenotypically on colonies identified as *E. coli* or *Salmonella* spp. isolated from each positive sample. If neither *E. coli* nor *Salmonella* spp. were identified among the three sub‐cultured colonies, the sample was declared negative. PCR was performed on colistin‐resistant colonies to confirm the *mcr* genes.

### Analysis of the results

To evaluate the performance of the method, specificity and sensitivity were calculated. Sensitivity and specificity of the enrichment step were calculated based upon the *mcr* PCR results. Sensitivity of the PCR was defined as the ability of the method to detect the *mcr* gene among the expected *mcr*‐positive samples. Specificity of the PCR was defined as the ability of the method to discard negative samples. Performance of the isolation step was expressed by sensitivity from results obtained with expected positive samples. Sensitivity of the isolation step was defined as the probability of the described screening procedure to selectively isolate the spiked colistin‐resistant strain from expected positive samples. The growth performance between media was evaluated statistically with a Fisher exact test with a *P* ≤ 0·05 for significant results.

## Conflict of Interest

No conflict of interest declared.

## Author Contributions

Agnès Perrin‐Guyomard, Sophie A. Granier, Jannice Schau Slettemeås, Kees Veldman contributed to conception and design of the study. Agnès Perrin‐Guyomard, Sophie A. Granier, Jannice Schau Slettemeås, Muna Anjum, Luke Randall, Manal AbuOun, Natalie Pauly, Alexandra Irrgang, Jens Andre Hammerl, Jette Sejer Kjeldgaard, Anette Hammerum, Alessia Franco, Magdalena Skarżyńska, Ewelina Kamińska, Dariusz Wasyl, Cindy Dierikx, Stefan Börjesson, Yvon Geurts, Marisa Haenni and Kees Veldman contributed to data acquisition. Agnès Perrin‐Guyomard and Marisa Haenni did analysis and interpretation of data. Agnès Perrin‐Guyomard, Marisa Haenni and Kees Veldman drafed the manuscript. Sophie A. Granier, Jannice Schau Slettemeås, Muna Anjum, Luke Randall, Manal AbuOun, Natalie Pauly, Alexandra Irrgang, Jens Andre Hammerl, Jette Sejer Kjeldgaard, Anette Hammerum, Alessia Franco, Magdalena Skarżyńska, Ewelina Kamińska, Dariusz Wasyl, Cindy Dierikx, Stefan Börjesson and Yvon Geurts did revision of the manuscript. All authors approved the final version of this manuscript.

## Funding

This publication is a part of the European Joint Programme One Health EJP. This programme has received funding from the European Union’s Horizon 2020 research and innovation programme under Grant Agreement No. 773830 and was also co‐financed by the involved institutions.

## Data Availability Statement

The data that support the findings of this study are available from the corresponding author upon reasonable request.

## Supporting information

Table S1: Confirmatory methods used by each participant
Table S2: Homogeneity and stability results
Table S3: Colony appearance according to the manufacturer instructions
Figure S1: Flow chart for the PCR step
Figure S2: Flow chart for the plating step
