# Supplementary material for: Multicentre evaluation of a selective isolation protocol for detection of mcr‐positive E. coli and Salmonella spp. in food‐producing animals and meat
Source: Lett Appl Microbiol. 2022 Apr 21;75(2):224–33. doi: 10.1111/lam.13717 (PMC9544698; doi:10.1111/lam.13717)
Supplement: Supplementary file 1 — Table S1: Confirmatory methods used by each participant Table S2: Homogeneity and stability results Table S3: Colony appearance according to the manufacturer instructions Figure S1: Flow chart for the PCR step Figure S2: Flow chart for the plating step [file LAM-75-224-s001.doc]

Table S1: Confirmatory methods used by each participant

| **Method** | **Laboratory code** | | | | | | | | | | | |
| --- | --- | --- | --- | --- | --- | --- | --- | --- | --- | --- | --- | --- |
|  |  | **A** | **B** | **C** | **D** | **E** | **F** | **G** | **H** | **I** | **J** | **K** |
|  |  |  |  |  |  |  |  |  |  |  |  |  |
| DNA extraction |  | PureLink Extraction Kit (Invitrogen) | DNeasy Blood & Tissue Kit (Qiagen) | DNeasy Blood & Tissue Kit (Qiagen) | DNeasy Blood & Tissue Kit (Qiagen) | Blood and Tissue Kit (Macherey Nagel) | DNeasy Blood & Tissue Kit (Qiagen) | QIAamp DNA Mini Kit (Qiagen) | DNeasy Blood & Tissue Kit (Qiagen) | DNeasy Blood & Tissue Kit (Qiagen) | DNeasy Blood & Tissue Kit (Qiagen) | DNeasy Blood & Tissue Kit (Qiagen) |
|  |  |  |  |  |  |  |  |  |  |  |  |  |
| *mcr* PCR |  | Rebelo *et al*, 2018 | Rebelo *et al*, 2018 | Rebelo *et al*, 2018 | In house | Lescat *et al*, 2018 | Rebelo *et al*, 2018 | Liu *et al*, 2016 and Xavier *et al*, 2016 | Rebelo *et al*, 2018 | Rebelo *et al*, 2018 | Rebelo *et al*, 2018 | Rebelo *et al*, 2018 |
|  |  |  |  |  |  |  |  |  |  |  |  |  |
| Bacterial identification |  | Maldi-Tof (Bruker) | Maldi-Tof (Bruker) | Maldi-Tof (Bruker) | Maldi-Tof (Bruker) | API 20E (bioMérieux) | PCR ( uspA: Chen et al, 1998; invA Stankievicius et al, 2006) | API 20E (bioMérieux) | Maldi-Tof (Bruker) | Maldi-Tof (Bruker) | Maldi-Tof (Bruker) | Maldi-Tof (Bruker) |
|  |  |  |  |  |  |  |  |  |  |  |  |  |
| Phenotypic confirmation |  | BMD* (Thermo Scientific™ Sensititre™) | BMD (Merlin) | BMD (Thermo Scientific™ Sensititre™) | BMD (Thermo Scientific™ Sensititre™) | Broth MIC (In house two fold dilution) | BMD (Thermo Scientific™ Sensititre™) | BMD (Thermo Scientific™ Sensititre™) | BMD  (Thermo Scientific™ Sensititre™) | BMD (Merlin) | BMD (Merlin) | BMD (Thermo Scientific™ Sensititre™) |

* BMD: broth microdilution

Table S2: Homogeneity and stability results

|  | | | Sample 1 (negative sample) |  | Sample 2 (*mcr-1*) |  | Sample 3 (*mcr-3*) |  | Sample 4 (negative sample) |  | Sample 5 (*mcr-4*) |  | Sample 6 (*mcr-5*) |
| --- | --- | --- | --- | --- | --- | --- | --- | --- | --- | --- | --- | --- | --- |
| Homogeneity |  | PCR | --- |  | +++ |  | +++ |  | --- |  | +++ |  | --- |
|  |  | Plating | --- |  | ++++ |  | ++++ |  | --- |  | ++++ |  | ---- |
|  |  |  |  |  |  |  |  |  |  |  |  |  |  |
| Stability |  | PCR | ND |  | +++ |  | +++ |  | ND |  | +++ |  | --- |
|  |  | Plating | ND |  | +++ |  | +++ |  | ND |  | +++ |  | --- |

(-) indicates negative result according to the number of aliquots per tested samples; (+) indicates positive result according to the number of aliquots per tested samples; ND: not determined

Table S3: Colony appearance according to the manufacturer instructions

| **Microorganism** |  | **Typical colony appearance** | | |
| --- | --- | --- | --- | --- |
|  |  | **CHROMID® Colistin R** | **CHROMagar^TM^ COL-*APSE*** | **COLISTIGRAM** |
| Escherichia coli |  | pink to burgundy | dark pink to reddish | metallic green sheen |
|  |  |  |  |  |
| *Salmonella* spp. |  | white or colourless | white or colourless | amber to colourless |


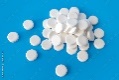

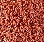

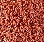

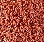


**1 g of caecal content (or 10 g of meat)**

**+ 9 ml BPW (or + 90 ml BPW)**

Pre-enrichment without colistin

Incubate at 37°C

3 hours +/- 1H

Selective enrichment with colistin

**Multiplex *mcr* PCR**


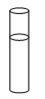


Mix gently

**+ 9 ml BPW**

**+ 2 discs of colistin 10mg**

**1ml pre-enrichment**

Incubate for 18-24h at 37°C

**1ml enrichment**

Genotypic detection

Store at 4°C


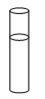

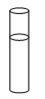

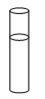

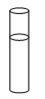

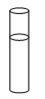

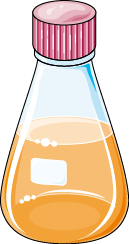

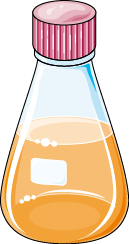

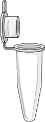

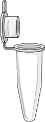

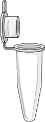

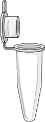

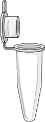

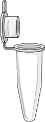


Figure S1: Flow chart for the PCR step

**Identification**

**Broth micro dilution**

**Multiplex *mcr* PCR**

Incubate for 18-24h at 37°C

Plating

Purification

Confirmation


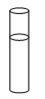


**CHROMagar^TM^**

**COL-*APSE***

**CHROMID®**

**Colistin R**

**COLISTIGRAM**

Positive samples stored at 4°C

**CHROMagar^TM^**

**COL-*APSE***

**CHROMID®**

**Colistin R**

**COLISTIGRAM**

Figure S2: Flow chart for the plating step
